# Are we there yet? A machine learning architecture to predict organotropic metastases

**DOI:** 10.1186/s12920-021-01122-7

**Published:** 2021-11-24

**Authors:** Michael Skaro, Marcus Hill, Yi Zhou, Shannon Quinn, Melissa B. Davis, Andrea Sboner, Mandi Murph, Jonathan Arnold

**Affiliations:** 1 Institute of Bioinformatics, University of Georgia, Athens, GA 30602 USA; 2 Department of Computer Science, University of Georgia, Athens, GA 30602 USA; 3 Department of Cellular Biology, University of Georgia, Athens, GA 30602 USA; 4 Caryl and Israel Englander Institute for Precision Medicine, New York Presbyterian Hospital-Weill Cornell Medicine, New York, NY 10065 USA; 5 Department of Pharmaceutical and Biomedical Sciences, University of Georgia, Athens, GA 30602 USA; 6 Weill Cornell Medicine, HRH Prince Alwaleed Bin Talal Bin Abdulaziz Alsaud Institute for Computational Biomedicine, New York, NY 10021 USA; 7 Department of Pathology and Laboratory Medicine, Weill Cornell Medicine, New York, NY 10065 USA; 8 Meyer Cancer Center, Weill Cornell Medicine, New York, NY 10065 USA

**Keywords:** Cancer, Metastatic organotropism, Machine learning, Transcriptomic profiling

## Abstract

**Background & Aims:**

Cancer metastasis into distant organs is an evolutionarily selective process. A better understanding of the driving forces endowing proliferative plasticity of tumor seeds in distant soils is required to develop and adapt better treatment systems for this lethal stage of the disease. To this end, we aimed to utilize transcript expression profiling features to predict the site-specific metastases of primary tumors and second, to identify the determinants of tissue specific progression.

**Methods:**

We used statistical machine learning for transcript feature selection to optimize classification and built tree-based classifiers to predict tissue specific sites of metastatic progression.

**Results:**

We developed a novel machine learning architecture that analyzes 33 types of RNA transcriptome profiles from The Cancer Genome Atlas (TCGA) database. Our classifier identifies the tumor type, derives synthetic instances of primary tumors metastasizing to distant organs and classifies the site-specific metastases in 16 types of cancers metastasizing to 12 locations.

**Conclusions:**

We have demonstrated that site specific metastatic progression is predictable using transcriptomic profiling data from primary tumors and that the overrepresented biological processes in tumors metastasizing to congruent distant loci are highly overlapping. These results indicate site-specific progression was organotropic and core features of biological signaling pathways are identifiable that may describe proliferative plasticity in distant soils.

**Supplementary Information:**

The online version contains supplementary material available at 10.1186/s12920-021-01122-7.

## Background

Metastasis accounts for 90% of cancer associated mortality [1]. While disease spread is a definitive turning point in patient pathology, metastasis is a long, arduous, and inefficient process for a primary tumor [1, 2]. To establish an overt colonization in a distant organ, metastasis proceeds through multiple restrictive bottlenecks. Tumor sheds must first retain membrane integrity during a violent intravasation and successfully navigate the circulatory vasculature. Arriving in the new settlement, cells must elude immune response, retain activation of growth signals, and survive radiotherapies or putative ablation via chemotherapeutics [3–5]. The possible organs sites of metastasis are tumor type specific; and in part determined by primary lesion anatomic location, intratumor metabolic reprogramming, augmented protein functions and disrupted biological pathways driving tumor cell fitness in the distant organs [6–10]. The dissemination of successful metastases is an organized process known as metastatic organotropism.

Metastatic organotropism is a long-standing problem in cancer research and characterizing the metastatic patterns of primary tumors is a critical step towards treating patients with advanced disease [11, 12]. Experimentally driven investigations have focused on characterizing the biological underpinnings of organotropic metastasis while computational approaches have developed tools attempting to predict the sites of metastases. Previous research has described the patterns of bone, liver, and lung tropisms. Bone tropisms arise primarily from breast and prostate cancers [13]. In prostate cancers, three major clusters of pathologies have evolved, one of which show high androgen receptor signaling and high bone-tropism compared to the other clusters [14, 15]. Liver tropisms primarily arise from breast, lung, and gastrointestinal cancers [13]. A 17-gene signature has been shown to indicate adverse outcomes for breast cancer patients and has some correlative evidence suggesting liver progression from breast tumors [16]. Lung tropisms are observed most commonly in breast, melanoma and thyroid cancers [13, 17]. Similar to liver tumors, a 54 gene panel expression signature has been developed for showing correlation for organotropic metastasis from breast tumors progressing to the lung [18].

Studies using molecular information for retrospective analyses of tumor metastatic sites have been xenograft selection studies that extrapolated organotropic features from metastasis microarray data. Studies leveraging RNA transcript profiling data have been designed for single tumor type progressing to a single site. We have found no significant study has been developed on classifying site-specific metastasis from human primary tumor transcriptomic profiling data [5, 19–28]. The most recent work investigating organotropic progression used no molecular data and instead used deep data mining of patient clinical data to model temporal patterns of tumor type site-specific progression and established a powerful co-occurrence based network but did not extract any biological determinants of tumor plasticity in distant organs [24].

Despite the significant progress made from previous modeling methods, a unified approach to predict site specific metastasis in multiple cancer types that learns the biological determinants of dissemination has not been resolved. We have leveraged the publicly available omics data and clinical annotations in the TCGA database to investigate metastatic organotropisms of multiple cancers. In this study, we build off the previous work and establish a machine learning architecture that models organotropic metastases by distinguishing the tumor type and in multiple cancer types predicts the loci of distant tumor metastases. We detail a migration from the canonical pipelines using differential expression for feature assessment and use statistical machine learning for feature selection to optimize classification. Our model systematically predicts site-specific metastases of primary tumors and our methods captured conserved core biological processes overrepresented in tumors of varying origin that seeded in concordant anatomic locations.

## Methods

### Review of data download of TCGA transcriptomic and clinical annotation data

The TCGA data portal has the clinical data commons that are publicly available for data mining in the clinical databank [29]. These data are accessible in multiple ways including Bulk/Batch API access, TCGA Biolinks software via Bioconductor, and Cart-Building on the portal website in a patient-by-patient search [29]. Currently, no unified patient disease progression information is directly available for bulk data mining on the portal website. Our progression annotation was built by text mining clinical files of progression annotations project by project using the batch query function in the TCGA Biolinks package. Each patient has multiple unique identifiers. In a project-by-project manner, each Case ID was cataloged. Each case ID query produced a case UUID that was used across the data types including the gene expression counts, VCF files, FASTQ files, images from slides, and clinical annotation for each experiment for each patient. Each UUID produces a patient summary. Each summary was broken down into: Data category, Experimental strategy, clinical annotations, and clinical supplemental files. The transcriptome counts files for each project were downloaded, normalized and analyzed. Each project has between 53 and 261 clinical annotation columns. The stringr and dplyr software packages were used for clinical annotation, data cleaning, and anatomical annotation [30]. Metastatic tumors identified in the clinical annotation file were drawn from the “metastatic tissue”, “sites of metastases” or “metastatic tissue site” column(s). Tumor progression labeled as “synchronous” were not included in the metastatic data as the clinical timeline of diagnosis was ambiguous. The diagnosis allows for tumors to be classified as synchronous ranging between the time of diagnosis up to 6 months following the diagnosis in varying tumor types.

### Review of synthetic sample generation

Synthetic samples were generated to balance positive and negative classes using the SMOTE algorithm; where positive classes were tumors that developed a metastasis in the tested location and negative classes were tumors that did not develop a metastasis in the tested locaiton [31]. Briefly, the Synthetic Minority Oversampling Technique (SMOTE) is an algorithm to increase the representation of a minority class in machine learning classification problems. The objective function for this approach sits on top of a distance based KNN algorithm. The synthetic oversampling technique begins by selecting a minority class instance. Then finds the instance’s k nearest neighbors. One of the minority class neighbors is chosen at random. A line is drawn between these two instances and a synthetic sample is generated along the line as a convex combination of the two real instances. This process repeats until it has created the desired number of synthetic samples. The number of synthetic samples generated was specific for each binary comparison. The authors suggest that the SMOTE algorithm can be used to generate a large sum of representative synthetic samples, however how large that sum is without over fitting the model is unknown. We employed an overfit prevention method during sample balancing. We measured 80% of the majority class and increased the representation of the minority to the match approximately 80% of the majority class rounded to the closest integer.

### Review of feature selection

Feature selection is a method in model building to reduce the dimensionality of a dataset. Overfitting can occur when the number of columns (features) outnumber the rows (instances) we can use for the model. To reduce the dimensionality of the problem we have employed three kinds of feature selection methods: Filter based, Wrapper-based and Embedded feature selection. Chi-square filtering calculates the chi-square metric between the target and the numerical variable and only reduces the features for the variables with the maximum chi-squared values. The SelectKBest, Chi2 and MinMaxScaler Libraries from Sklearn and feature_selection module were used [32]. A Recursive feature selection estimator iteratively reduced the dimensionality of the data set by recursively considering smaller and smaller subsets of each feature block. The RFE was trained on each initial block of features and the importance of each feature was obtained through the feature_importances attribute. The RFE and LogisticRegression libraries from Sklearn and feature_selection module were used [32]. For embedded methods, Random forest classifier, random forest regression and lasso regression with a logistic regression estimator and L1 penalty were employed. These algorithms have an embedded feature selection method to stratify and rank features. The SelectFromModel, RfC and RfR libraries were imported from Sklearn [32, 33]. We cross validated these approaches by extracting support values in each using the get_support methods, summing the true feature support Booleans for each feature in each block across all five methods and sorting features by selection support.

Iterative Feature selection was conducted by splitting the 60,483 transcript features into 100 blocks of approximately 600 features to be assessed by the above algorithms. We extracted support values for each feature from each selection method. Each block was assessed independent of all other blocks in each classification. Transcripts were filtered for features that showed the highest cross-validated support in multiple or all algorithms. Dimensionality was finally reduced by filtering out co-linear features. The top 10% of highest scoring features were kept from each block for a total number of approximately 5000 candidate transcripts (50 transcripts × 100 blocks). The remaining transcripts were used as the input features in each binary classification. Tree-based models were selected as the best fit for the classification to account for the variability in number selected features in each classification and to allow model attributions to be extracted post-hoc.

### Review of model building

Random Forest classification and Gradient boosted tree classifiers were built to classify site specific progression from primary tumors. The selected features in each binary classification were used as input attributes into model classification. The model is set to report rounded value for classification but is capable of posterior probability for class likelihood. The code and the pretrained models are available through the documented Github. Model building and usage is documented on the Github wiki page.

### Review of feature recapture

Feature recapture was the final phase of model building and analysis. Testing the statistical significance of feature recapture in independently generated lists following bioinformatic analysis is an indirect however well documented technique to determine non-random enrichment [34]. Two sets of feature recapture were analyzed and displayed in Additional file 1: Table S7. The tests were conducted; within cancer class seeding loci and the between cancer classes metastasizing in matching locations. The Fisher’s exact was used to evaluate the significance of recapture between lists, as the significance of deviation from the null hypothesis can be directly calculated. Our null hypothesis was that the feature recapture when analyzing matched seeding locations across cancer types was by chance; therefore, no biological meaning can be drawn from the phenomena. Our alternative hypothesis was that recapture of features within class and between matching seeding locations indicates similar distant metastatic potential and offers candidate biomarkers for organotropic metastasis, respectively. The contingency table was set as; the background of the search space for the information gain algorithm. The starting feature selection space for each classification was the entire human transcriptome. As all of the binary compassions initially began considering all 60,483 transcripts, and each set of selected features were independently generated, the total transcriptome remained the background for all tests. In list A of each contingency table, we place the top 1000 features for each classification of primary tumor seeding location. In list B, we assess a second primary tumor type and/or metastatic location feature list. We test the significance of the intersection of the two lists considering the list sizes, background and overlap in contingency table. The GeneOverlap package on Bioconductor was used to conduct the Fisher’s exact tests [35].

### Gene set overrepresentation and semantic analysis

The clusterprofiler package was used to conduct an overrepresentation test in the GO database [36]. The selected features for each metastatic location in each cancer type were translated into their associated GO biological process IDs using the bitr function in the clusterProfiler package [36]. The overrepresented GO biological pathways were passed to into the GoSemSim package and simplify enrichment package [37]. A similarity matrix of biological functions was made using the simplfyEnrichment package in R [38]. A heatmap was produced by clustering the similarity scores of the biological functions using the package default binary cut function. A Fisher’s exact test was conducted using the base GeneOverlap in R [35]. The background was changed from the human transcriptome to the GO database to account for the change in the search space [39]. The UpsetR package in R was used to display the bar graph of overlapping biological processes in the tumors seeding in matched locations [40]. All overlaps were tested between cancers metastasizing in similar organs.

### Data availability and code

We used public data sets drawn from the TCGA database using the GDC data commons for this project and its analyses [41, 42]. We have provided all the custom computer code to produce these models.

Our code is currently available for view and use in a public Github repository: https://github.com/michaelSkaro/Classification_of_organotropic_metastases. The docker image containing all relevant environment variables, dependencies and a demo test data set is also made publicly available on docker hub and integrated into the Github actions. We have a documented wiki page that is available, demonstrating the installations, displays visualization and describes script usage within the pipeline. We have provided a general usage script that runs the entire metastatic classification pipeline. At the command line it can be ran using the metastasis_pipeline.py script within the built docker container. We have provided a general usage feature selection pipeline Feature_selection.py. We have provided the organotropic features sets for all cancer types selected in this study in the Additional file 1: data tables. We have provided all enrichment and recapture code in the source code.

## Results

### Classification of tumor type

**Fig. 1:**
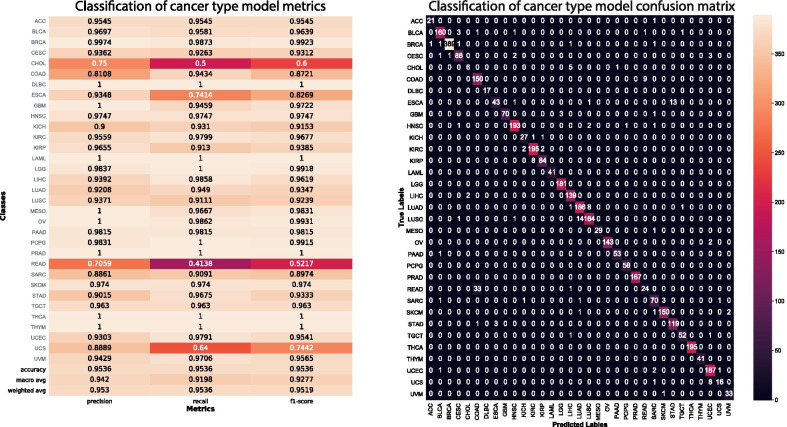
Classification of tumor type. Classification of Cancer type. The confusion matrix detailing sample type specific performance for the GBT classification of tumor transcriptomes. 33 cancer types were considered by the model as annotated by their four letter TCGA code. The scale bar on the right-hand vertical axis denotes the density for each tile where dark tiles indicate low number of predicted values and red/white values indicate high numbers of predicted values. The major diagonal denotes the cancer type match between predicted and true labels where true labels are annotated along the left-side vertical axis and predicted labels are annotated across the horizontal axis

Each tumor type is unique and potential metastatic sites of progression are limited based on the tumor gene expression profile, anatomic location, and blood circulation [24]. We hypothesized that each tumor type has subsets of features associated with tissue specific progression. Therefore, classifying tumor type was considered a critical step towards extracting patterns of organotropic metastasis. Thirty-three tumor types were considered by the model and are annotated by their four-letter code in the tumor type column in all figures and tables. Figure 1 displays the confusion matrix of the model as a heatmap and displays the model precision, recall and f1-score with normalized performance for population size classifying 33 cancer types in the TCGA database. Our model performs in the excellent range on thirty of the cancer classes, Cholangiocarcinoma (CHOL) showed the worst performance as the population of 45 was too small to develop a strong model for cancer type classification. Esophageal carcinoma and stomach adenocarcinoma showed some misclassification in between the types, given these tumors have been shown to be pathologically very similar in previous research this was unsurprising [43]. Colorectal adenocarcinoma (COAD) showed considerable misclassification specifically misidentifying COAD for Renal adenocarcinoma (READ) and vice-versa. The COAD and READ classes are combined in the UCSC genome browser database, and combined COAD and READ in further analyses as the metastatic progressions showed a considerable overlap.

**Fig. 2:**
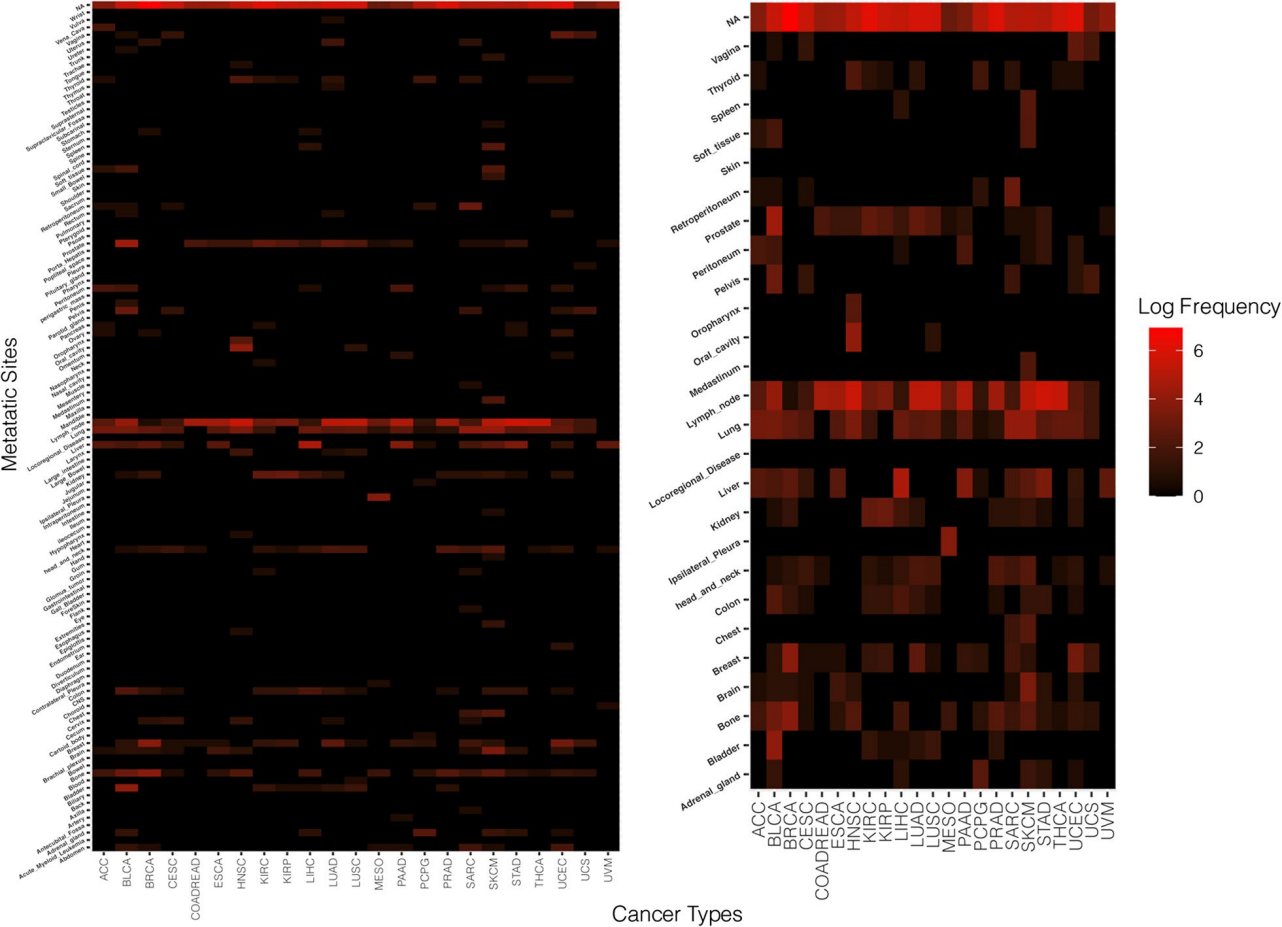
Observed sites of metastatic progression in the TCGA database. Thirty-three cancers in the TCGA database have recorded RNA sequencing data. Within twenty-three projects 125 anatomic locations have clinically annotated metastatic progression. Unique metastatic sites of progression found within the population are annotated on the vertical axis. The cancer type four letter codes are annotated on the horizontal axis. The heatmaps are stratified by log frequency of occurrence in the data set. The right heatmap are were locations with the greatest frequency amongst all sites. COAD and READ have been combined in this section of the analysis

Overall, the cancer type classification model performed in the excellent range with a macro average precision of 94.2, macro average recall of 91.98 and macro average F1 score of 92.77. The classified results were used to carry forward for site specific metastases prediction. The classification of the primary tumor type significantly decreased the complexity of predicting possible sites of metastatic progression for each primary tumor. We annotated 125 metastatic locations in the ten thousand patient samples separated in twenty-three TCGA projects containing transcriptomic and clinical data (Fig. 2). The most observed sites of metastasis were Bone, Liver, Lung and Lymph Node (Fig. 2). We filtered for metastatic sites with at least eight clinical annotations of progression for a given site and an overall total population of over fifty patients with documented non-synonymous progression of disease arising from the primary tumor. Following filtering we were able to analyze 35 tumor metastatic site pairs.

### Classification of organotropic progression

Thirty-three cancer types in TCGA were analyzed in this study, based on the availability of annotated metastatic progression in the TCGA clinical data. For sixteen cancer types, we predicted site specific organotropic metastases. The classification of the organotropic metastases in the sixteen cancer types occurred in three phases. First, synthetic sample generation, followed by feature selection, and finally classification of progression. Synthetic sample generation was used to increase the representation of tumors that metastasized to each of the tested locations. Feature selection was used to reduce the dimensionality of the data and to find transcripts that best separated the tumors that metastasized to a tested locations from negative cases. We combined five feature selection algorithms to assess feature value discriminating between positive and negative classes in each classification independent of all other comparisons [44].

**Fig. 3:**
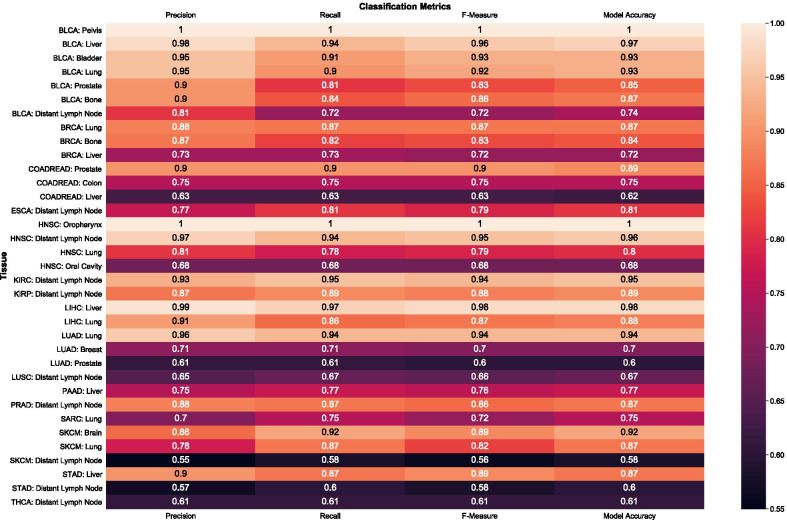
Prediction of Site-specific Metastases. Displayed are the model performance metrics predicting site specific metastasis. The data was classified following a train test split where 30% of the annotated transcriptome population were held out. The performances reported are on out of bag instances that were not used as synthetic templates for training. Model performances are reported on a scale of 0 to 1. Cancer type label are in the four-letter code from the TCGA database. Total support are instances in the test set where a positive class was observed are reported in Additional file 1: data tables

In Fig. 3 we show the performance of classification in sixteen cancer types. We report four metrics for the classification of site-specific progression in each cancer; precision, recall, F1 Measure and Model Accuracy. We observed an overall average precision of 0.82, average recall of 0.82, average F1 Measure of 0.82 and average accuracy of 0.82 considering all sites and all predictions. We performed in the excellent range on twenty six of 35 classification pairs. The projects with the fewest errors were the larger projects; Bladder cancer, Breast cancer, Colorectal cancers, and lung cancers. Sites with the strongest model support for prediction were Bone, Liver, Lung and Lymph Node. Cancer type specific performance is detailed in Table 1. Considering all progressions for each cancer type.

**Table 1:** Average model metrics by cancer

TCGA-Project	Avg. Precision	Avg. Recall	Avg. F-Measure	Avg. Model accuracy
BLCA	0.93	0.87	0.89	0.90
BRCA	0.82	0.80	0.81	0.81
COADREAD	0.76	0.76	0.76	0.75
ESCA	0.77	0.81	0.79	0.81
HNSC	0.86	0.85	0.85	0.86
KIRC	0.93	0.95	0.94	0.95
KIRP	0.87	0.89	0.88	0.89
LIHC	0.95	0.91	0.93	0.93
LUAC	0.76	0.75	0.75	0.75
LUSC	0.65	0.67	0.66	0.67
PAAD	0.75	0.77	0.76	0.77
PRAD	0.88	0.87	0.86	0.87
SARC	0.70	0.75	0.72	0.75
SKCM	0.73	0.79	0.76	0.79
STAD	0.73	0.74	0.74	0.74
THCA	0.61	0.61	0.61	0.61

Displayed are the cumulative model performance metrics aggregating all locations for each cancer type. The cancers are labeled with their four letter TCGA code. Model metrics reported right to left were classification precision, classification recall, classification F-Measure and classification accuracy. Model performance variance and standard deviation are reported in the Additional file 1. Positive and Negative class specific performance reported in Additional file 1: data tables

**Fig. 4:**
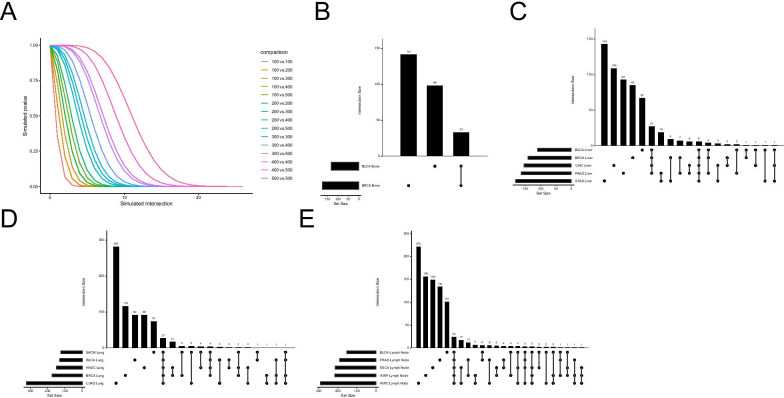
Simulated and observed overrepresented GO biological processes. Gene set enrichment analysis was conducted using the clusterProfiler package in R. The Go ontology database was used to investigate feature enrichment in Biological Processes for each metastatic location in each cancer type that was classified by the model. The upsest plots were generated using the UPsetR package. The bars represent the GO IDs with an adjusted *p* value < 0.05 after Bonferroni correction. **A** Simulated enrichment of randomly selected transcript features overrepresented in GO. **B** Enriched processes in Bone metastases. **C** Enriched processes in Liver metastases. **D** Enriched processes in Lung metastases. **E** Enriched processes in Lymph Node metastases. Statistical significance and GO:ID enrichment results included in Additional file 1: data tables

After the classification of the organotropic metastases, we predicted tumors metastasizing to congruent loci may exhibit similar biological changes in the primary tumor endowing proliferative plasticity in the distant organ locations. To this end, we used the top 1000 selected features from each feature selection to conduct pathway enrichment. In Fig. 4A. We simulated the number of expected biological processes to overlap if 1000 randomly selected transcripts were enriched in the GO database. It is known that Ensemble transcript IDs map to multiple GO biological process IDs and therefore there is a high probability of false discovery due to random chance. To establish that our observed overlap between lists of GO BP IDs were significant, we modified previously published gene overlap protocols and conducted a weighted simulation of our feature selection methods where IDs with the least amount of mapping match GO IDs are given priori over IDs with many matches [31]. The weighted simulation was conducted by randomly selecting two sets of 1000 transcript features, conducting a GO over representation test within each list, filtering for significantly overrepresented processes in the feature sets followed by testing the simulated overlap of the two independently generated GO:ID lists. We conducted this simulation a total of 750,000 times using 50,000 simulations for each possible intersection combination. We tested all pairwise combinations of 5 possible lengths of GO:ID lists ranging from 100 GO:IDs to 500 GO:IDs. The simulated results are stratified by the colored lines in Fig. 4A. Our simulation shows that the feature selection method consistently produced significantly higher overlap than in random simulation. In Fig. 4B–E we show the number of overrepresented biological processes in the tumors metastasizing to bone, liver, lung, and Lymph Node, respectively. We reported the list overlaps, odds ratio and adjusted p.value after Bonferroni adjustment in the Additional file 1: Table S7.

**Fig. 5:**
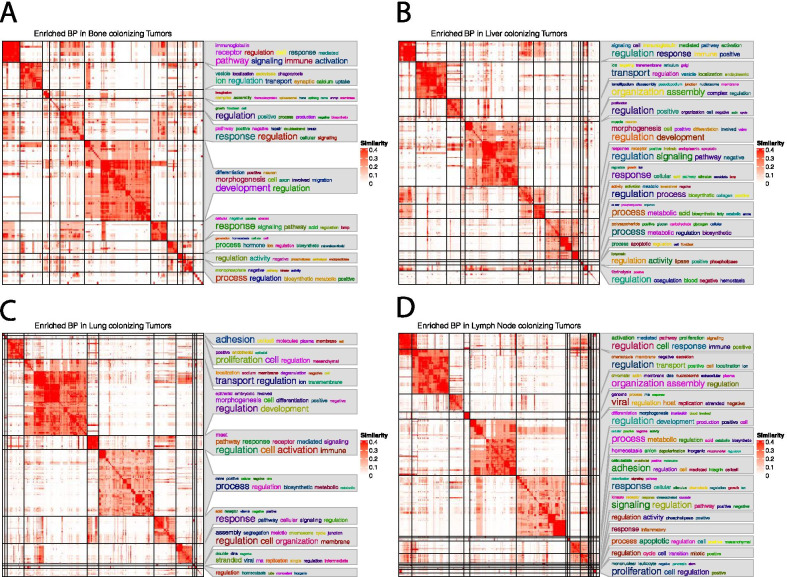
Shared significantly overrepresented biological processes. Gene set enrichment analysis was conducted using the clusterProfiler package in R. The Go ontology database was used to investigate feature enrichment in Biological Processes for each metastatic location in each cancer type that was classified by the model. SimplifyEnrichment package was used to cluster the semantic similarity between shared overrepresented biological processes in tumors metastasizing to concordant locations. **A** Enriched processes in Bone metastases. **B** Enriched processes in Liver metastases. **C** Enriched processes in Lung metastases. **D** Enriched processes in Lymph Node metastases. Statistical significance and GO:ID enrichment results included in Additional file 1: data tables. Similarity scores are on a scale of 0 to 1

In Fig. 5A–D we cluster the sematic similarity of the GO:ID terms that passed the selection and filtering. We display four heatmaps that describe the biological processes found to be overrepresented in primary tumors metastasizing to concordant locations. The largest cluster common among all the comparisons was regulation of morphogenesis and migration. This is a significant result as collective cell migration is a hallmark of metastatic cancer and further suggests a progressive tumors may be identified by the expression profiles [45].

## Discussion

The capacity to accurately determine the site-specific metastases of patients’ primary tumors is directly applicable to clinical actions for patients. Following tumor resection; transcriptomic analysis of a patient’s tumor can provide valuable insight into disease progression and can aid clinician’s treatment interventions [46]. We present an accurate and precise machine learning architecture that can classify the tumor type and can identify if and where a primary tumor will metastasize. Embedded in our model we offer potential users the opportunity to report the locations of the metastases and additionally retain the posterior probabilities of metastatic progression to each location. This offers users the ability to integrate investigation specific calibration for their data and report the confidence of the classification in the clinical setting.

The model improves on previous work in two fundamental ways. The model increases the scope and performance comparison to previous work modeling either a single cancer type or single metastatic location and identifies biological feature determinants of organotropic metastasis from unified transcript profiling data. The model was shown to be broadly applicable in 16 different cancer types. Our feature selection method is uncommon amongst canonical bioinformatics or biomedical pipelines. The differentiation of the positive class feature space was only discernable from the negative class feature space following statistical machine learning centered feature selection methods. The features that are represented in the Additional file 1: data tables were produced cross validating five feature selection method and extracting model attribution support for the best features in each comparison.

Our model is not without clear limitations. By breaking down a multi-label, multi-output experiment into NxM binary classification experiments we sacrificed detecting possible features that may be present in non-mutually exclusive progression. An example of this break down occurs when one patient’s tumor metastasized to the liver and the lung. The model will fail to find features that may be dictating the multi-organ expansion of the patient’s disease. We justify this sacrifice with an opportunity cost. While we will not find these coalescent features as there are not enough coalescent cases to properly model these phenomena, we do produce a model with very high sensitivity and specificity to detect if and where both metastases will arise in a given case. Further, the model is built in a way, upon receipt of more data, we can make the necessary modifications from a binary comparisons list to an All vs. All classification. The transition to an All vs. All classification presents the clear second limitation of this model; the very costly overhead of data production. Our model relies on the largest ever unified conglomerate of tumor transcriptome data to produce the level of precision and recall we achieved on only 16 cancer types of the 33 TCGA projects we investigated. This model is reliant on the high-quality data production pipeline in TCGA. The transcript profiling data for each tumor were produced from sequencing of patient tumors of extremely high purity which is very uncommon in most studies. If this model is to be broadly incorporated into the medical community it will need a very deep and diverse set of transcriptomes to train on that is much larger than our current TCGA dataset.

### Next steps

Our next steps will be to include more cancer types. As the publicly available data continue to grow as a super set of TCGA and the International Cancer Genome Consortium (ICGC), more projects will have clinically annotated tumor and normal transcriptomes. Further, the TCGA database documentation has become more unified and is continuously growing in its clarity. This will allow us to incorporate multiple data types into a multiomic approach that may illuminate genetic, genomic, epigenetic and transcriptomic features working to provide proliferative plasticity in metastatic soils. Finally, if the public data grows by a significant margin, we can approach characterizing organotropic metastasis with an All vs. All model.

## Conclusion

Our machine learning architecture expands the understanding of the cancer metastasis. The leading cause of cancer associated death is metastatic progression of disease, however incorporating this tool into the clinical timelines for patients may offer clinicians opportunities for pre-metastatic therapeutic interventions. We demonstrate our model can detect if and where metastases will arise. Our methods of synthetic sample generation and feature selection produced a clear and concise biological data-based model of metastatic progression in multiple tumor types. Our recaptured features are offered as candidate biomarkers of site-specific metastatic organotropism.

## Supplementary Information

**Additional file 1.** **MOT data tables. Table S1:** Tumor vs. Normal metrics: For six tumor types we show the model can detect tumor vs. normal transcriptomic expression profiles. **Table S2:** Site Specific classification: Site specific metastatic classification metrics. **Table S3:** Positive and Negative metrics for T/N transcriptomes site specific metastases. **Table S4:** All binary classification confusion matrices. **Table S5:** Top 1K selected features for site specific metastases predictions. **Table S6:** Enriched GO IDs in organotropic metastases. **Table S7:** Intersection of overrepresented GO processes measured by Fisher’s exact test.

## Data Availability

The datasets analysed in this study were obtained from the TCGA database using custom computer code that is available on https://github.com/michaelSkaro/Classification_of_organotropic_metastases.

## Abbreviations

TCGA: The Cancer Genome Atlas
UUID: A universally unique identifier
VCF: Variant call files
SMOTE: Synthetic Minority Oversampling Technique
KNN: K-Nearest Neighbor
GO: Gene Ontology
ACC: Adrenocortical Carcinoma
BLCA: Bladder Urothelial Carcinoma
BRCA: Breast Ductal Carcinoma
CESC: Cervical Carcinoma
CHOL: Cholangiocarcinoma
COAD: Colorectal Adenocarcinoma
ESCA: Esophageal Carcinoma
GBM: Glioblastoma Multiforme
HNSC: Head and Neck Squamous Cell Carcinoma
LIHC: Hepatocellular Carcinoma
KIRC: Kidney Chromophobe Carcinoma
KICH: Kidney Clear Cell Carcinoma
KIRC: Kidney Papillary Cell Carcinoma
LGG: Lower Grade Glioma
LUAD: Lung Adenocarcinoma
LUSC: Lung Squamous Cell Carcinoma
MESO: Mesothelioma
OV: Ovarian Serous Adenocarcinoma
PAAD: Pancreatic Ductal Adenocarcinoma
PCPG: Paraganglioma & Pheochromocytoma
PRAD: Prostate Adenocarcinoma
SARC: Sarcoma
SKCM: Skin Cutaneous Melanoma
TGCT: Testicular Germ Cell Cancer
THYM: Thymoma
THCA: Thyroid Papillary Carcinoma
UCS: Uterine Carcinosarcoma
UCEC: Uterine Corpus Endometrioid Carcinoma
UVM: Uveal Melanoma
ICGC: International Cancer Genome Consortium
